# Human Movement Detection and Idengification Using Pyroelectric Infrared Sensors

**DOI:** 10.3390/s140508057

**Published:** 2014-05-05

**Authors:** Jaeseok Yun, Sang-Shin Lee

**Affiliations:** Embedded Software Convergence Research Center, Korea Electronics Technology Institute, 25 Saenari-ro, Bundang-gu, Seongnam 463070, Korea; E-Mail: sslee@keti.re.kr

**Keywords:** pyroelectric infrared sensor, human movement detection, human idengification, machine learning, occupancy sensing, occupant localization

## Abstract

Pyroelectric infrared (PIR) sensors are widely used as a presence trigger, but the analog output of PIR sensors depends on several other aspects, including the distance of the body from the PIR sensor, the direction and speed of movement, the body shape and gait. In this paper, we present an empirical study of human movement detection and idengification using a set of PIR sensors. We have developed a data collection module having two pairs of PIR sensors orthogonally aligned and modified Fresnel lenses. We have placed three PIR-based modules in a hallway for monitoring people; one module on the ceiling; two modules on opposite walls facing each other. We have collected a data set from eight subjects when walking in three different conditions: two directions (back and forth), three distance intervals (close to one wall sensor, in the middle, close to the other wall sensor) and three speed levels (slow, moderate, fast). We have used two types of feature sets: a raw data set and a reduced feature set composed of amplitude and time to peaks; and passage duration extracted from each PIR sensor. We have performed classification analysis with well-known machine learning algorithms, including instance-based learning and support vector machine. Our findings show that with the raw data set captured from a single PIR sensor of each of the three modules, we could achieve more than 92% accuracy in classifying the direction and speed of movement, the distance interval and idengifying subjects. We could also achieve more than 94% accuracy in classifying the direction, speed and distance and idengifying subjects using the reduced feature set extracted from two pairs of PIR sensors of each of the three modules.

## 1. Introduction

With the advancement of sensor and actuator technologies, our indoor environment, such as buildings, has been instrumented with various sensors, including temperature, humidity, illumination, CO_2_ and occupancy sensor, and, thus, can be aware of changes in the user's state and surrounding, finally controlling building utilities to adapt their services and resources to the user's context, e.g., automatic lighting control, heating, ventilation and air-conditioning (HVAC) system adjustment, electrical outlet turn-off, unusual behavior detection and home invasion prevention. Such context-aware systems have deployed occupant location as the principal form of the user's context. Accordingly, indoor tracking and localization is one of the key technologies for providing activity-aware services in a smart environment.

Pyroelectric infrared (PIR) sensors are well-known occupancy detectors. They have been widely employed for human tracking systems, due to their low cost and power consumption, small form factor and unobtrusive and privacy-preserving interaction. In particular, a dense array of PIR sensors having digital output and the modulated visibility of Fresnel lenses can provide capabilities for tracking human motion, idengifying walking subject and counting people entering or leaving the entrance of a room or building. However, the analog output signal of PIR sensors involves more aspects beyond simple people presence, including the distance of the body from the PIR sensor, the velocity of the movement (*i.e.*, direction and speed), body shape and gait (*i.e.*, a particular way or manner of walking). Thus, we can leverage discriminative features of the analog output signal of PIR sensors in order to develop various applications for indoor human tracking and localization.

In this paper, we present an empirical study of human movement detection and idengification using PIR-based modules having two pairs of orthogonally-aligned PIR sensors. We have developed a data collection module consisting of two pairs of PIR sensors whose dual sensing elements are orthogonally aligned and Fresnel lenses are modified to narrow the field of view of the PIR sensors to its horizontal motion plane, a data logger, op-amp circuits and a rechargeable battery. We have placed three PIR-based modules in a hallway for monitoring people; one PIR-based module is placed on the ceiling; two PIR-based modules are placed on opposite walls facing each other. We have collected a data set from eight experimental subjects when walking in three different conditions: two directions (back and forth), three distance intervals (close to one wall sensor, in the middle, close to the other wall sensor) and three speed levels (slow, moderate, fast). We have employed two types of feature sets: a raw data set and reduced feature set composed of amplitude and time to peaks; and passage duration extracted from each PIR sensor. We have performed classification analysis according to various configurations, including the number of modules involved (ceiling-mounted module *vs.* wall-mounted modules), the number of PIR sensors involved (a single PIR sensor, a pair of PIR sensors orthogonally aligned and two pairs of PIR sensors orthogonally aligned), the feature set (raw data set *vs.* reduced feature set) and well-known machine learning algorithms, including instance-based learning and support vector machine. Our findings show that with the raw data set captured from a single PIR sensor of each of the three modules, we were able to achieve more than 92% correct detection of direction and speed of movement, the distance interval and idengification of walking subjects. We could also achieve more than 94% accuracy in classifying the direction, speed level and distance interval and idengifying walking subjects using the reduced feature set extracted from each of the three modules equipped with two pairs of PIR sensors.

The rest of the paper is organized as follows: Section 2 introduces various indoor localization and tracking and motion detecting systems using PIR sensors. Section 3 presents a human movement detection and idengification system and explains what aspects of PIR sensors we employ to detect the direction and speed of movement and the distance interval. Section 4 describes the PIR sensor-based movement detecting device and data collection procedure and explains which features we extract from the raw data set and which classifiers we employ for machine learning. Section 5 presents the experimental results of the classification analysis with the raw data set and reduced feature set extracted from the raw data set collected. Section 6 discusses remaining challenges for our methods. Finally, Section 7 offers concluding remarks.

## 2. Related Work

### 2.1. PIR Sensor-Based Applications in Smart Environments

PIR sensors are commonly used with a variety of sensors in diverse applications for building smart environments, such as healthcare, smart energy system and security. Han *et al.* presented an occupancy and indoor environment quality sensing method based on a suite of sensors, including PIR sensors, CO_2_ sensors, humidity sensors and concentration sensors [1]. Tsai *et al.* illustrated a way of reducing the standby power consumption of lighting devices based on a PIR sensor, an ambient light sensor and lighting duration modules [2]. They also demonstrated a way of reducing the standby power consumption of a personal computer monitor in sleep status [3]. Erickson *et al.* implemented a power-efficient occupancy-based energy management system based on camera-based and PIR sensor-based wireless sensor networks for opportunistically controlling HVAC system and, thus, increasing energy efficiency, while maintaining conditioning effectiveness [4]. This capability of PIR sensor-based occupancy and motion detection for diverse application domains provided the motivation for this research into the feasibility of a human movement detection system using various features extracted from the PIR sensor signal.

### 2.2. Indoor Human Tracking with PIR Sensors

Many researchers have devoted a great deal of effort to developing localization technology for indoor human tracking using PIR sensors. Gopinathan *et al.* developed a pyroelectric motion tracking system based on coded apertures, which could detect human motion in one of the 15 cells in a 1.6-m square area using four PIR detectors [5]. Shankar *et al.* developed a human tracking system using a low-cost sensor cluster consisting of PIR sensors and Fresnel lens arrays to implement the desired spatial segmentations [6]. They analyzed the response characteristics of the sensor cluster and extracted the velocity and the direction of motion over large areas of over 12 m. Hao *et al.* presented a human tracking system using an MSP430 family microcontroller, an RF transceiver and a radial sensor module with eight PIR detectors with Fresnel lens arrays arranged around a circle [7]. They showed that the system can be used to track a single human target by detecting its angular displacement while moving. Luo *et al.* demonstrated a 3D simulation study for human tracking using PIR sensors [8]. Their approach showed the visibility modulation of each sensor detector and the layout of sensor modules and proposed algorithms for localizing and tracking people based on the binary output of the PIR sensors.

### 2.3. Human Movement Detection with PIR Digital Outputs

More specifically, researchers have been working on detecting movement direction and counting people entering or leaving the entrance of a room or building using the on-off output signal of PIR sensors. Hashimoto *et al.* presented a people counting system composed of a one-dimensional eight-element custom-fabricated array detector, an IR-transparent lens and an oscillating mechanical chopper [9]. They show a 99% recognition accuracy of the two-way back-and-forth moving direction and a 95% recognition accuracy of the number of passersby. Wahl *et al.* developed a people counting system for an office space based on self-sustaining ultra-low power sensor nodes composed of a pair of uni-directional PIR sensors [10]. They simply differentiate the direction of movement at a gateway by observing the time difference between inward-facing and outward-facing PIR sensors. Through a real-life office room study, they show their prototype system performs well on detecting all occupant movements to and from the office room with a very low error rate (<1%). They also proposed an approach to estimate people count per office space using distributed pairs of PIR sensors and an algorithm to process the distributed sensor information [11].

### 2.4. Human Movement Detection with PIR Analog Outputs

Lee proposed a novel method of detecting the motion direction for an object moving in the field of view of a single PIR sensor, whose dual sensing elements are reversely polarized and aligned in the motion plane of the PIR sensor [12]. As Lee presented, the analog output signal of PIR sensors involves more aspects beyond simple on-off triggering, and such features have been exploited in several ways for recognizing motion direction. Zappi *et al.* built a low-cost PIR sensor-based wireless network system for detecting the direction of movement and distinguishing the number of people (two and three) walking in line, as well as walking side by side in a hallway [13]. They showed the 100% correct detection of direction of movement and 89% correct detection of the number of people. The authors also built a cluster system composed of two PIR sensors facing each other in a hallway for detecting the direction of movement and distance intervals (close to one sensor, in the middle, close to the other sensor) when a person is walking [14,15]. They extracted the passage duration and output amplitude from the PIR output signals and performed classification analysis using the support vector machine and *k*-nearest neighbor algorithms. The experimental results show 100% correct detection of the direction of movement and 83.49%–95.35% correct detection of distance intervals. More recently, Yun and Song presented a novel method of detecting the relative direction of human movement in eight directions uniformly distributed with two pairs of PIR sensors whose sensing elements are orthogonally aligned [16]. With the raw data sets captured from two orthogonally–aligned PIR sensors with modified lenses, they achieved more than a 98% correct detection of the direction of movement. They also found that with the reduced feature set composed of three peak values in the time domain for each PIR sensor, they were able to achieve 89%–95% recognition accuracy according to machine learning algorithms.

### 2.5. Human Idengification with PIR Sensors

Many researchers have also been working on a human idengification system using PIR sensors. Fang *et al.* presented a human idengification system using a PIR sensor whose visibility is modulated by a Fresnel lens array and a principal components regression method [17]. They investigated the performance of the idengification system in terms of the optimal element number of the lens array, the height of the sensor location, the sensor-to-object distance and walking speed. Fang *et al.* presented a method for idengifying subjects walking through a dependent and an independent path using PIR sensors with modulated visibilities and hidden Markov models [18]. The average recognition rates for 10 persons are 91% and 78.5% in the path-dependent and path-independent cases, respectively. They also demonstrated a biometrics system for open-set walker recognition using a PIR sensor, a Fresnel lens array and the maximum likelihood principal components estimation (MLPCE) method [19]. Hao *et al.* introduced a wireless distributed PIR system for tracking and idengifying multiple humans based on their body heat radiation and gait [20]. For a single walker idengification, the experiment for five persons with sensor fusion and four PIR modules, each of which is composed of eight PIR sensors, shows 100% recognition accuracy. Tao *et al.* presented a person localization algorithm using an infrared ceiling sensor network for providing various personalized services in an office environment [21]. The result shows 84% accuracy on recognizing five persons with support vector machine. Recently, Sun *et al.* presented a multi-target, complex scenario idengification system based on a binary PIR-based sensor network [22].

However, the previous research for human idengification exploited the digital on-off output signals of PIR sensors with visibility modulation, and it is worth investigating the availability of analog output signals of PIR sensors for idengifying subjects, as in the research on classifying the walking direction and speed and the distance of the body from the sensor. To our knowledge, there is no published literature investigating human idengification based on the analog output signals of PIR sensors and machine learning algorithms. We also consider various sensor configurations, such as the number of sensors involved and different sensor placement, including ceiling-mounted and wall-mounted ones, to achieve high recognition accuracy in human movement detection and idengification. In addition, we perform an extensive study using a larger number of classification algorithms than previous ones, helping people choose appropriate algorithms for their PIR-based applications.

## 3. Detecting Human Movement and Sensors

### 3.1. Human Movement Detection and Idengification

**Figure 1.**
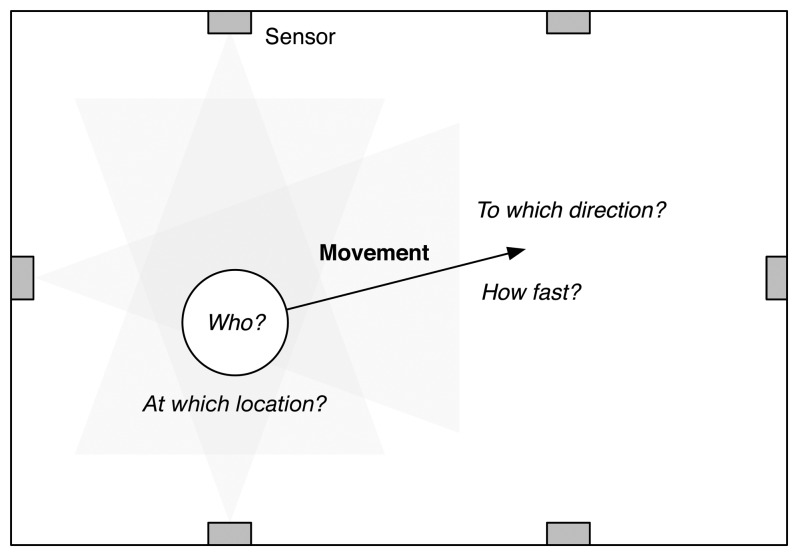
Human movement detection and idengification for indoor person tracking.

In order to build a smart environment where systems could understand the activities a user is involved with and the surroundings and then adapting their services and resources to the user's context, we need to develop a robust motion detection and tracking system using various sensors. Many researchers have devoted their efforts toward building robust motion tracking systems using vision-based sensors, *i.e.*, cameras. The research projects based on vision-based sensors mainly consider position, speed, direction, shape and size (*i.e.*, the number of pixels in cameras) as the principal context for idengifying the users and understanding their activities [23]. Accordingly, as shown in Figure 1, a motion tracking system will need to robustly detect: (1) the identity of the moving object; (2) at which location the object is; (3) to which direction the object is moving; and (4) how fast the object is moving. We next consider the sensing systems for human movement detection and idengification for building a robust motion tracking system.

### 3.2. Pyroelectric Infrared Sensors

Sensing systems for human movement detection and idengification collect a raw data set from the human body and extract distinguishable features to recognize the principal context that we mentioned previously: the object location, the direction of the movement, the speed of the movement and the identity of the object. Numerous sensing systems have been studied using various sensors, including cameras, motion sensors, pressure pads, radars, electric field sensors. Among them, in this paper, we focus on pyroelectric infrared (PIR) sensors as sensing systems to detect human body movement in an indoor environment.

PIR sensors are well known and have been widely used as a simple, but powerful presence trigger for alarms, such as surveillance systems and automatic lighting control. In particular, PIR sensors could address the invasion of privacy issues raised by the use of camera-based surveillance systems. Whatmore presented an extensive survey on the technology of pyroelectric materials, devices and its potential applications [24]. PIR sensors belong to the class of thermal IR detectors, *i.e.*, voltage change-thermoelectrical conversion, and use materials having a pyroelectric effect, e.g., LiTaO_3_, which is spontaneously polarized in the crystal structure [25]. Although PIR sensors have been commonly used as simple presence triggers using digital output, the analog output signal of PIR sensors depends on several aspects, including the direction and speed of a moving object, the distance of the body from the PIR sensor, the body shape and the presence of multiple people.

**Figure 2.**
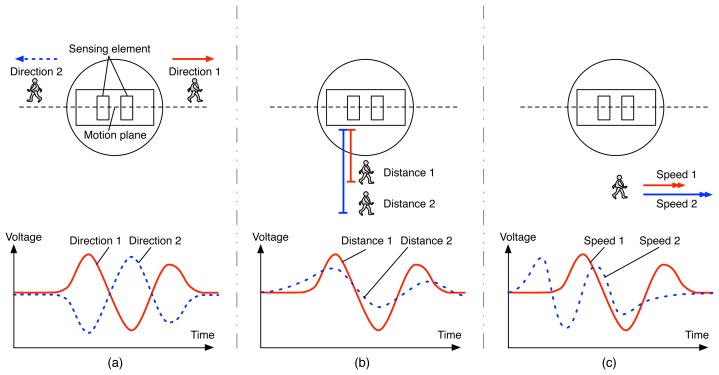
A schematic presentation of a pyroelectric infrared (PIR) sensor with dual sensing elements aligned in a motion plane and its output signal when walking: (**a**) the output signal in the case of walking in different directions; (**b**) the output signal in the case of walking at different distances; (**c**) the output signal in the case of walking at different speed levels.

Figure 2 is a schematic presentation of a PIR sensor having two sensing elements aligned in a motion plane and its output signal captured in the case of walking: (1) in different directions; (2) at different distances; and (3) at different speed levels. The graph from Figure 2a shows that by alternating the polarities of the sensing elements in a PIR sensor, we can discriminate between the two signals captured according to the relative directions of walking, *i.e.*, left to right and right to left, and subsequently to obtain the clear direction information of the moving object. Similarly, the graphs from Figure 2b,c show that by using the different output signal patterns (e.g., amplitude and time to peaks), we can discriminate between the two signals captured according to the distances of the human body from the PIR sensor and the speed levels of the walking human, *i.e.*, close/far and slow/fast; and subsequently, to obtain the clear distance and speed information of the moving object. Finally, with all these aspects, we can imagine a way of idengifying the moving object based on a human movement detecting device composed of a set of PIR sensors. Therefore, the main result of this paper is to demonstrate a way of detecting human movement and idengifying subjects based on a human movement detecting device composed of a set of PIR sensors and to discuss the experimental results according to direction, distance and speed estimation for human movement and, finally, human idengification.

## 4. A PIR-Based Movement Detecting Device

### 4.1. PIR Module and DAQ System

As Yun and Song proposed in [16], two pairs of PIR sensors whose sensing elements are orthogonally aligned could classify walking directions into eight categories (*i.e.*, N, NW, W, SW, S, SE, E, NE). For our research, we also assume that the signals collected from the orthogonally-aligned PIR-based detecting device are good enough to distinguish between the distances of the human body from the PIR sensors and the speeds of the moving body.

**Figure 3.**
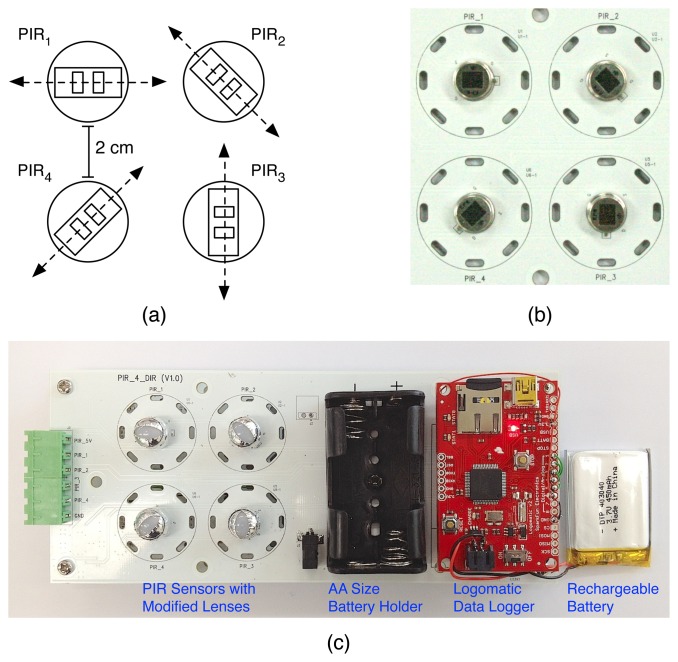
**(a)** A schematic diagram of a set of four PIR sensors whose sensing elements are each oriented at W-E, NW-SE, S-N and SW-NE angles; (**b**) a top view of four PIR sensors we implemented; (**c**) a top view of the human movement detecting device consisting of PIR sensors, a Logomatic v2 Serial SD data logger from SparkFun Electronics, a rechargeable battery and a custom-fabricated data acquisition board integrated with op-amp circuits.

Accordingly, for our experiments, we have created a human movement detecting device based on two pairs of PIR sensors orthogonally aligned, as shown in Figure 3. The human movement detecting device consists of two pairs of PIR sensors, a Logomatic v2 Serial SD data logger from SparkFun Electronics, a rechargeable battery and a custom-fabricated data acquisition board. Figure 3a shows a schematic diagram of a set of four PIR sensors whose sensing elements are each oriented at W-E, NW-SE, S-N and SW-NE angles, and Figure 3b is a top view of the four PIR sensors we have implemented. PIR sensors are separated by a 2-cm space considering the Fresnel lens setup, which is able to shape the field of view of PIR sensors. We have used IRA-E710 PIR sensors from Murata Manufacturing Co., whose vertical and horizontal field of views are both 90° [26]. Because IRA-E710 PIR sensors output a low analog voltage signal, we have developed op-amp circuits to amplify the low PIR output signal. To acquire amplified PIR sensor signals, we have created a printed circuit board (PCB) having PIR sensors and op-amp circuits, whose outputs are connected to the analog inputs of a Logomatic v2 Serial SD data logger. The Logomatic v2 SD data logger is configured to record each analog input as a time series on a 2 GB micro-SD memory card, with a sampling rate of 10 Hz. It is already known that a 10-Hz sampling rate shows the best performance in human activity analysis based on PIR sensor-based systems [21]. A rechargeable battery can continuously power four PIR sensors and the data logger during data collection. We can also power the data collection module with two AA batteries.

PIR sensors are used in conjunction with Fresnel lenses to shape their field of view [27]. Fresnel lenses can be manufactured by molding plastic materials having transmission characteristics appropriate for a particular wavelength range, e.g., the human body (8–14 *μ*m). For our experiments, instead of using multi-zone Fresnel lenses mainly employed in traditional intruder detection systems [28], we used single-zone Fresnel lenses, IML-0637, from Murata Manufacturing Co. [26]. As presented in [16], we assume that by narrowing the field of view of PIR sensors to its horizontal motion plane aligned with the sensing elements, we could reduce the signal captured from walking in the other directions, and this would help in distinguishing the walking direction aligned with the sensing elements from the other directions, thus improving recognition accuracy. With the assumption, we have shielded IML-0637, single-zone Fresnel lenses with metallic foils, as shown in Figure 3c.

### 4.2. Experimental Setup and Data Collection

**Figure 4.**
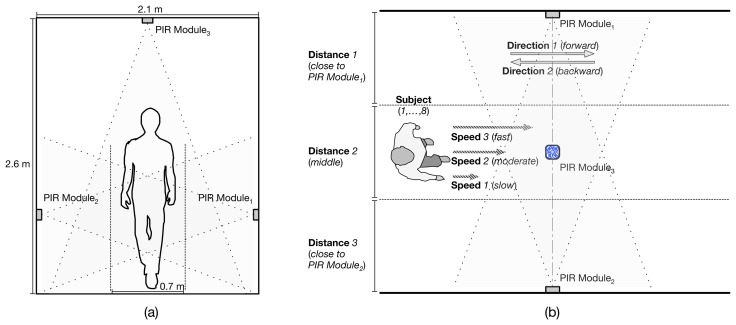
Experimental setup for data collection while an experimental subject is walking, (**a**) a front view of the monitored field; (**b**) a top view of the monitored field.

Our experiments consisted of capturing PIR sensor signals while experimental subjects are walking through a monitored field. A hallway in our office building is chosen as a monitoring field for collecting PIR sensor signals. Of course, office rooms or larger public areas could be considered, but the PIR sensor-based human movement detecting device mounted on walls (probably, the ceiling-mounted one is good for collecting data sets) may not collect data sets appropriate for our analysis due to the long distance of the body from the PIR sensor. Accordingly, we selected an indoor scenario, *i.e.*, monitoring people walking through a hallway in our building. The hallway is 2.1 m wide, and the ceiling is 2.6 m high. For data collection, we have placed three PIR-based modules that we developed in the hallway; one module on the ceiling; two modules on opposite walls facing each other, as shown in Figure 4a. The ceiling-mounted PIR-based module is attached to the ceiling in the middle of the field. Two wall-mounted PIR-based modules are attached on opposite walls facing each other, at a height of 0.8 m from the floor. It has been already shown that a set of PIR sensors and Fresnel lenses located at a height of 0.8 m presents the best performance for path-dependent human idengification [18].

We have collected PIR sensor signals from eight experimental subjects. The average height of the subjects is 170.67 cm, and the standard deviation is 5.22 cm. The average weight of the subjects is 69.22 kg, and the standard deviation is 8.32 kg. Each participant is asked to walk through the monitored field equipped with three PIR-based modules in three different conditions: direction, walking in two different directions (*i.e.*, forward and backward); distance, walking at three different distances from PIR modules (*i.e.*, close to PIR Module_1_, middle, close to PIR Module_2_); speed, walking at three different speed levels (*i.e.*, slow, moderate, fast), as shown in Figure 4b. All subjects are asked to walk as normally as possible within the range of their daily walking style. For each experimental condition—direction, distance, speed—we have captured 10 walking samples from each participant and, finally, collected a total of 1,440 (= 8 subjects × 2 directions × 3 distance intervals × 3 speed levels × 10 passages) walking samples.

### 4.3. Data Analysis and Feature Extraction

**Figure 5.**
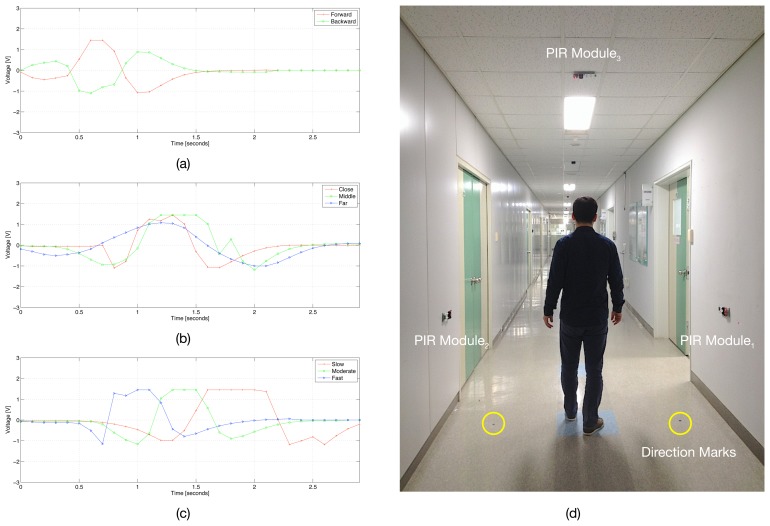
The output signals captured from the PIR_1_ sensor of the wall-mounted Module_1_ in the case of different experimental conditions, and a photo of an experiment for data collection: (**a**) the PIR output signal in the case of walking forward and backward; (**b**) the PIR output signal in the case of walking at three different distances; (c) the PIR output signal in the case of walking at three different speed levels; (**d**) an experiment for collecting PIR signals when walking.

Figure 5 shows the signals captured from a PIR sensor (*i.e.*, PIR_1_ in Figure 3) of the wall-mounted data collection module (*i.e.*, PIR Module_1_ in Figure 4) and a photo of an experiment for data collection. Figure 5a–c shows the captured output signals of a PIR sensor in the case of different experimental conditions, *i.e.:* (1) walking forward and backward; (2) walking at three different distance intervals (close, middle, far); (3) and walking at three different speed levels (slow, moderate, fast), respectively. From each figure, we can know that the captured PIR signals vary according to the different experimental conditions, as we explained in Figure 2. Accordingly, we will be able to use these quite different patterns of the PIR output signals in the case of different experimental conditions and leverage them to detect the aspects of human movement, including direction, distance interval and speed level, and, finally, idengify the subject.

Because it is clear to see that there are quite different patterns between PIR output signals captured in different experimental conditions, we could employ a raw data set consisting of the time series captured from the PIR-based modules as a feature set for classifying directions, distances and speeds and idengifying experimental subjects. In this case, assuming that we use all four PIR sensors of each of the three PIR-based modules, and a walking sample is 3 s, a feature set for a walking sample is composed of 360 amplitude values (= 3 PIR-based modules × 4 PIR sensors/PIR-based module × 3 s × 10 values/s).

**Figure 6.**
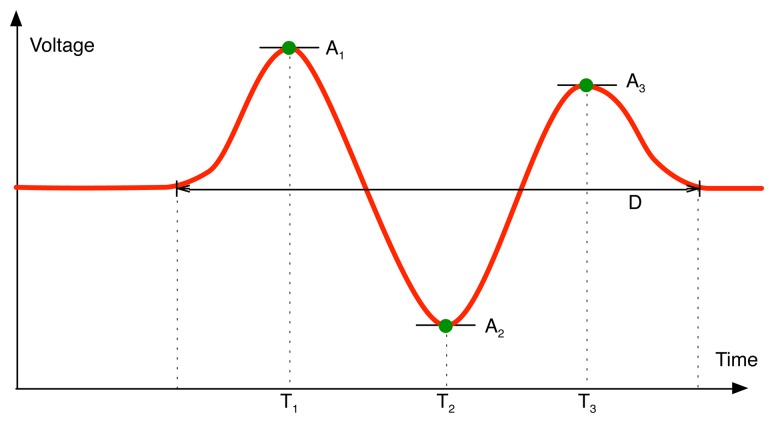
Feature extraction, amplitude (A_1_, A_2_, A_3_) and time (T_1_, T_2_, T_3_) to three peaks and passage duration (D).

However, it is already known that the signal amplitude (the difference between the maximum and minimum value of the PIR output) and the passage duration (the time during the PIR output exceeds custom thresholds) can be used as a feature set to classify the distance of movement from the PIR sensors [15]. They also used a peak detection method (one positive and one negative) to recognize the direction of movement. It is also presented that the amplitude values of three peaks in PIR signal can be used as a feature set for classifying walking directions [16]. Accordingly, as shown in Figure 6, we have decided to employ a reduced feature set consisting of amplitude (A_1_, A_2_, A_3_) and time (T_1_, T_2_, T_3_) to three peaks and passage duration (D) for a PIR sensor for our classification analysis. In this case, assuming that we use all four PIR sensors of each of three PIR-based modules, a feature set for a walking sample is composed of 84 features (= 3 PIR-based modules × 4 PIR sensors/PIR-based module × 7 features/PIR sensor).

Even though we can build a reduced feature set for our classification analysis, we might be able to further reduce features by removing redundant PIR sensors or modules. That is, without employing all three PIR-based modules or all four PIR sensors in each module, we might be able to achieve reasonable performance for classification analysis, finally reducing the computational cost and memory requirement for training and classifying data sets. We will demonstrate the effect of the trade-off between the amount of computation load and recognition accuracy in the result sections below.

### 4.4. Classifiers

Among various available machine learning algorithms, we chose seven classification methods: Bayes net, decision tree (C4.5), decision table, instance-based learning (*k*-nearest neighbor algorithm), multilayer perceptron, naive Bayes and support vector machine. Support vector machine is chosen as one of the state-of-the-art discriminative methods with a good performance in many applications. For support vector machine, we use three types of kernels, including linear, quadratic and cubic. We chose the simple *k*-nearest neighbor algorithm from instance-based learning algorithms and the decision tree and decision table from rule-based learning algorithms. In addition, Bayes net is chosen as one of generative models to show its performance in our experiments. We also used naive Bayes and multilayer perceptron as classifiers. All the experiments based on these classifiers were carried out using Weka developed by the Machine Learning Group at University of Waikato [29]. Weka is a data mining tool with open source machine learning software in Java. It supports all the classification methods we chose for classification. Therefore, we would be able to compare the experimental results among various classifiers and feature sets. We also used Eclipse to build and run a Java program based on the Java classes supported by Weka [30].

## 5. Experiments and Results

In our experiments, we used the 1,440 walking samples collected from eight subjects in different walking conditions, *i.e.*, two different directions (back and forth), three different speed levels (slow, moderate and fast) and three different distance intervals (close to one sensor, in the middle, close to the other sensor), as explained in Section 4.2. For each experiment, we used all 1,440 walking samples combined with 10 times 10-fold cross-validation, in other words, 10 different 10-fold cross-validation experiments with the same learning method and data set, averaging 100 experimental results. In the experimental result, we summarize the average recognition accuracy, but we do not show the standard deviation of the results, because the average values are very close to each other (*i.e.*, very small standard deviation) for all classifiers. We have performed classification analysis with two types of feature sets: raw data set and reduced feature set. In Section 5.1, we first present the experimental result with the raw data set, *i.e.*, the time series captured from the PIR-based modules for classifying walking direction, distance and speed and idengifying subjects; and, then, in Section 5.2, we demonstrate the classification result with the reduced feature set explained in Section 4.3, composed of amplitude and time to peak values and passage duration extracted from the PIR sensor signals.

### 5.1. Classification Analysis with the Raw Data Set

In this section, we demonstrate the experimental result with the raw data set, *i.e.*, the time series captured from the PIR-based modules for classifying walking directions (backward and forward), distance intervals (close, middle, far), speed levels (slow, moderate, fast) and idengifying subjects.

#### 5.1.1. Classifying Directions with the Raw Data Set

Table 1 summarizes the experimental result for classifying walking directions (*i.e.*, forward and backward) over the selected classification methods based on PIR signals. In Table 1, we can know that the instance-based learning method (*k*-nearest neighbor algorithm) and Bayes net show the best classification performance. This result is not surprising, because the past works in [15,16] performed the recognition of motion direction with the *k*-nearest neighbor algorithm and show a good recognition accuracy. Support vector machine, in particular, with linear kernel and multilayer perceptron, also show good performance. The other algorithms show almost or over 98% recognition accuracy, except naive Bayes.

Note that classifying directions with a single (PIR_1_) sensor of each of all three modules performs as well as the other cases, *i.e.*, using a pair of PIR sensors orthogonally aligned (PIR_1_ and PIR_3_) or two pairs of PIR sensors orthogonally aligned (PIR_1_ and PIR_3_, PIR_2_ and PIR_4_). Therefore, we can know that a single PIR sensor of each PIR-based module will be enough to achieve a good performance in the two-way back-and-forth walking direction recognition. Now, we need to perform an investigation into the recognition performance with respect to the number of modules involved.

Table 2 summarizes the experimental result for classifying walking directions with respect to the number of modules involved based on PIR_1_ signals. The result with two wall-mounted PIR-based modules facing each other (*i.e.*, Module_1_ and Module_2_) shows 100% correct detection of walking direction, as also presented in [15]. More surprisingly, the results with a single module, *i.e.*, a single PIR sensor on the ceiling or walls, performs more than a 99% correct detection of the back-and-forth walking direction, probably because the PIR_1_ sensor of every module (and, thus, the sensing elements in the PIR sensor) is aligned with the motion plane of walking.

#### 5.1.2. Classifying Distances with the Raw Data Set

Table 3 summarizes the experimental result for classifying walking distances (*i.e.*, close to PIR Module_1_, middle, close to PIR Module_2_). In Table 3, we can know that instance-based learning method (*k*-nearest neighbor algorithm) and Bayes net show the best classification performance. This is very similar to the result obtained in walking direction classification and presented in the past work of [15]. Support vector machine, in particular, with quadratic and cubic kernels and multilayer perceptron, also show good performance, though those algorithms may require more computational load for training data sets than the *k*-nearest neighbor algorithm.

Similar to the result of walking direction classification, a single PIR sensor of each PIR-based module will be enough to achieve a good performance. Thus, we perform an investigation into the recognition performance with respect to the number of modules involved. Table 4 summarizes the experimental result for classifying walking distances with respect to the number of modules. The result with two wall-mounted PIR-based modules facing each other (*i.e.*, Module_1_ and Module_2_) shows about a 95% correct detection of walking distance intervals. The results with a ceiling-mounted module (*i.e.*, Module_3_) show a performance of more than 97% correct detection of walking distance intervals (even better than two wall-mounted modules), thus increasing the recognition accuracy (>99%) when using all three PIR-based modules, including wall- and ceiling-mounted modules.

#### 5.1.3. Classifying Speeds with the Raw Data Set

Table 5 summarizes the experimental result for classifying walking speed levels (*i.e.*, slow, moderate, fast). In Table 5, we can know that the instance-based learning method (*k*-nearest neighbor algorithm) shows again the best classification performance. Support vector machine, in particular, with the quadratic and cubic kernels, also shows good performance for walking speed classification.

Similar to the result of classifying walking directions and distance intervals, a single PIR sensor of each PIR-based module will be enough, and thus, we perform classification analysis with respect to the number of modules involved. Table 6 summarizes the experimental result for classifying walking speed levels with respect to the number of modules. The result with two wall-mounted PIR-based modules facing each other (*i.e.*, Module_1_ and Module_2_) shows about 92% correct detection of walking speed levels. The result with a ceiling-mounted module (*i.e.*, Module_3_) performs more than a 90% correct detection of walking speed, thus increasing the recognition accuracy (about 95%) when using all three PIR-based modules, including wall- and ceiling-mounted modules.

#### 5.1.4. Idengifying Subjects with the Raw Data Set

Table 7 summarizes the experimental result for idengifying subjects. In Table 7, we can know that the instance-based learning method (*k*-nearest neighbor algorithm) outperforms all other algorithms and shows the best classification accuracy. Support vector machine with quadratic and cubic kernels is the second best algorithm.

Unlike previous classification analysis, such as distance, direction and speed, the more PIR sensors are involved, the better recognition accuracy could be achieved. However, the experimental result with a single (PIR_1_) sensor of each of all three modules also shows a good performance (about 92%); thus, this would be a trade-off between recognition accuracy and the computational load required.

We also perform classification analysis with respect to the number of modules involved. Table 8 summarizes the experimental result for idengifying walking subjects with respect to the number of modules. The result with two wall-mounted PIR-based modules facing each other (*i.e.*, Module_1_ and Module_2_) shows about an 87% correct idengification of walking subjects. The result with a ceiling-mounted module (*i.e.*, Module_3_) does not show a good performance (about 63%), and thus, we can conclude that a human movement detecting system composed of only ceiling-mounted PIR sensors without wall-mounted ones may not idengify walking subjects well.

### 5.2. Classification Analysis with the Reduced Feature Set

In this section, we demonstrate the experimental result with the reduced feature set explained in Section 4.3, composed of amplitude and time to peaks and passage duration extracted from PIR sensor signals for classifying walking directions (backward and forward), distance intervals (close, middle, far), speed levels (slow, moderate, fast) and idengifying subjects.

#### 5.2.1. Classifying Directions with the Reduced Feature Set

In Section 5.1.1, we have presented a ceiling- or wall-mounted module equipped with a single PIR_1_ sensor can detect two-way, back-and-forth walking directions with more than 99% accuracy. We have also demonstrated the algorithms appropriate for walking direction classification, including Bayes net, instance-based learning, multilayer perceptron and support vector machine with a linear kernel. Accordingly, in this section, we present the experimental result of classifying walking directions with the reduced feature set and the machine learning algorithms that have shown a good performance in the experiments based on the raw data sets. Table 9 summarizes the experimental result for classifying walking directions based on the reduced feature sets with respect to the modules and features, such as amplitude and time to peaks. Regardless of the classification algorithms involved, the results with amplitude values without time information show more than 97% accuracy, except when using only the ceiling-mounted module, Module_3_. This is probably because narrowing the field of view of PIR_1_ by aluminum foil to its horizontal motion plane aligned with the sensing elements may reduce the signal captured (*i.e.*, the amplitude of peaks), in particular, when the subjects are walking within Distance 1 or 3 (see Figure 4). Accordingly, we can conclude that for walking direction classification, a single PIR sensor placed on a wall and its amplitude values of peaks will be enough to achieve a good performance, but in case of a ceiling-mounted PIR sensor, we need to use both amplitude and time to peaks.

#### 5.2.2. Classifying Distances with the Reduced Feature Set

In Section 5.1.2, we have presented that a ceiling- or a pair of wall-mounted modules equipped with a single PIR_1_ sensor works well for classifying distance intervals, *i.e.*, about 95% accuracy. We have also demonstrated the algorithms appropriate for walking distance classification, including Bayes net, instance-based learning, multilayer perceptron and support vector machine with quadratic and cubic kernels. Accordingly, we perform classification analysis based on the machine learning algorithms and the reduced feature set. Table 10 summarizes the experimental result with respect to the modules and features. Overall, we can know that instance-based learning shows the best performance, in particular, when using all three modules and both amplitude and time to peaks as features.

#### 5.2.3. Classifying Speeds with the Reduced Feature Set

In Section 5.1.3, we have presented that a ceiling- or a pair of wall-mounted modules equipped with a single PIR_1_ sensor works well for classifying speed levels, *i.e.*, about 90% accuracy. We have also demonstrated the algorithms appropriate for walking speed classification, including instance-based learning and support vector machine with quadratic and cubic kernels. Accordingly, we perform a classification analysis based on the machine learning algorithms and the reduced feature set. In this study, we have also employed passage duration as a feature, because it might depend highly on the subject's walking speed. Table 11 summarizes the experimental result with respect to the modules (*i.e.*, a ceiling-mounted module *vs.* a pair of wall-mounted modules) and PIR sensors (*i.e.*, a single PIR sensor *vs.* all four PIR sensors). Overall, support vector machine with cubic kernel shows the best performance, in particular, when involving a pair of wall-mounted modules and all four PIR sensors, though there is no big difference between the accuracy with and without the ceiling-mounted module (*i.e.*, Module_3_).

#### 5.2.4. Idengifying Subjects with the Reduced Feature Set

In Section 5.1.4, we have presented that a pair of wall-mounted modules facing each other, each of which has a single PIR sensor, could achieve about an 87% recognition accuracy for idengifying eight subjects. We have also presented that all three modules, each of which has a single PIR sensor, could achieve about a 92% recognition accuracy. We have also demonstrated that the recognition accuracy could be increased (until 95%) as the number of PIR sensors involved in the classification is increased. In addition, we have presented the algorithms appropriate for walking subject idengification, including instance-based learning and support vector machine with quadratic and cubic kernels. Accordingly, we perform classification analysis based on the machine learning algorithms and the reduced feature sets. Table 12 summarizes the experimental result with respect to the modules and PIR sensors. Overall, we can know that support vector machine with quadratic and cubic kernels shows the best performance (about 96%, even better than the result with the raw data set), in particular, when using all PIR sensors equipped in all modules. It should be also noted that the result without a ceiling-mounted PIR-based module (*i.e.*, Module_3_) shows more than a 91% recognition accuracy in idengifying walking subjects.

## 6. Discussion

From the experimental results, we can know that a pair of PIR-based modules mounted on opposite walls facing each other could classify the direction of movement, the distance of the body from the PIR sensors and the speed level of movement during two-way, back-and-forth walking and even idengify the walking subjects. A ceiling-mounted PIR-based module could also classify the direction, distance and speed of walking subjects, but does not perform subject idengification well. Accordingly, we can imagine extensions to this study adapted to building a smart environment, where a set of PIR sensors are attached on opposite walls facing each other at the entrance to the room and multiple PIR-based modules are distributed in a square grid across the ceiling in the room. In the smart environment, the wall-mounted PIR sensors at the entrance could idengify the user entering the room, and the ceiling-mounted PIR-based modules could detect the user's movement, including direction, distance and speed, robustly tracking the user and, thus, helping the system build a rich model of the user's context.

The experimental results using only a single PIR sensor (*i.e.*, PIR_1_) embedded in each module have shown good performance in classifying directions, distances and speeds, and this is probably because the walking samples we have used in our experiments are collected from two-way, back-and-forth walking, and PIR_1_ (and thus, its sensing elements) that each of the PIR-based modules is equipped with, is well aligned with the motion plane, *i.e.*, the walking directions. However, we also found that the more PIR sensors, *i.e.*, a pair of PIR sensors orthogonally aligned (PIR_1_ and PIR3) or two pairs of PIR sensors orthogonally aligned (PIR_1_ and PIR3, PIR2 and PIR4), could improve the recognition accuracy, in particular, in the experiment for idengifying walking subjects. In addition, for other public spaces at home or work, where occupants perform multi-directional movements, ceiling-mounted PIR-based modules rather than wall-mounted PIR-based modules are appropriate for human movement detection, and of course, the more the number of PIR sensors that are involved in the modules, the better the performance becomes.

In this study, we have performed an investigation into the recognition accuracy with respect to various machine learning algorithms, including Bayes net, decision tree (C4.5), decision table, instance-based learning (*k*-nearest neighbor algorithm), multilayer perceptron, naive Bayes and support vector machine with linear, quadratic and cubic kernels. Among them, Bayes net works well for classifying walking directions and distance levels. Multilayer perceptron also works well for classifying walking speeds, as well as walking directions and distance intervals. However, instance-based learning (*k*-nearest neighbor algorithm) and supper vector machine, in particular, with quadratic and cubic kernels outperform all other algorithms in most analyses. Between them, instance-based learning might be a better solution, because support vector machine with quadratic or cubic kernels usually requires a greater computation load for the training data set, though instance-based learning probably requires larger memory resources to retain a set of classified (*i.e.*, labeled) examples for performing supervised learning. Nevertheless, for the study for idengifying subjects, in particular, using the reduced feature set, support vector machine with quadratic or cubic kernels may be the only solution for good performance (see Table 12).

As presented in Section 4.3, the computation cost for human movement detection and idengification using the devices we have implemented will vary according to the number of modules (Module_1_, Module_2_, Module_3_), the number of PIR sensors with which the modules are equipped (PIR_1_, PIR_2_, PIR_3_, PIR_4_) and the features (raw data set or reduced feature set composed of amplitude and time to peaks, and passage duration) involved in the classification analysis. In the cases of a pair of wall-mounted modules facing each other, we could achieve almost or more than 85% recognition accuracy for classifying walking directions, distances and speeds based on the reduced feature set collected from a single PIR_1_ sensor with which each module was equipped. In the study, we have used 14 features (= 2 PIR-based modules × 1 PIR sensor/PIR-based module × 7 features/PIR sensor). In the case of a ceiling-mounted module, we could achieve almost or more than an 84% recognition accuracy for classifying walking directions, distances and speeds based on the reduced feature set collected from a single PIR_1_ sensor. In the study, we have used only seven features (= 1 PIR-based module × 1 PIR sensor/PIR-based module × 7 features / PIR sensor). However, for idengifying subjects with more than a 90% recognition accuracy, we need to employ all four PIR sensors with which each wall-mounted module is equipped, as presented in Table 12.

Our system shows a similar performance (>99%) to the previous PIR sensor-based systems in classifying walking directions and distance intervals. For idengifying walking subjects, our system shows better performance (96.56%) than the previous PIR sensor-based idengification methods (e.g., 91% in [18], 84% in [21]) except Hao's work (100%) in [20]. However, we cannot claim that our PIR sensor-based idengification system will perform better (or even worse) than other PIR-based systems, because the number of subjects idengified are different (e.g., eight subjects for our study vs. five subjects for Hao's work in [20]), and the data set for idengification might be collected in different walking conditions. As a consequence, although there would still be a question of which method is better in terms of recognition accuracy, we can conclude with confidence that the analog output signals of PIR sensors installed on the ceiling and walls can be used to reliably classify walking direction, the distance of the human body from the sensor and the speed level and to idengify walking subjects in indoor environments.

## 7. Conclusion

We have presented a human movement detecting system based on pyroelectric infrared (PIR) sensors and machine learning technologies for classifying the direction of movement, the distance of the body from the PIR sensors, the speed of movement during two-way, back-and-forth walking and idengifying the walking subject. To collect PIR sensor signals, we have built a PIR-based module consisting of two pairs of PIR sensors orthogonally aligned, op-amp circuits, a data logger and a rechargeable battery. We have placed three PIR-based modules: one module mounted on the ceiling and two modules mounted on opposite walls facing each other in a hallway. Using the PIR-based modules, we have collected PIR sensor signals when eight different experimental participants were walking through the monitoring field in three different conditions: direction, walking in two different directions (*i.e.*, forward and backward); distance, walking at three different distances from PIR modules (*i.e.*, close to PIR Module_1_, middle, close to PIR Module_2_); speed, walking at three different speed levels (*i.e.*, slow, moderate, fast). Based on the data set collected from the PIR-based modules, we have performed classification analysis for detecting walking direction, distance, speed and the subject using two types of feature sets: a raw data set and a reduced feature set composed of amplitude and time to peaks and passage duration for each PIR sensor signal, and various machine learning algorithms, including instance-based learning and support vector machine. Our results show that it is feasible to detect the direction and speed of movement and the distance of the body from the PIR sensors; and idengifying subjects with more than a 92% recognition accuracy using the raw data set collected from a single PIR sensor of each of the three PIR-based modules. We could also achieve more than a 94% accuracy in classifying the direction, distance and speed and idengifying subjects using the reduced feature set from two pairs of PIR sensors of each of the three PIR-based modules.

## Figures and Tables

**Table 1.** Comparison of recognition accuracy (%) of classifying walking directions based on the raw data set with respect to the number of PIR sensors of all modules.

**Classifier**	**Recognition Accuracy (%)**		
	Module_1,2,3_ 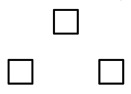		
	PIR_1_ 	PIR_13_ 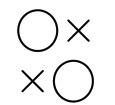	PIR_12,3,4_ 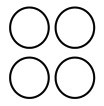
Bayes Net	**99.99**	99.99	99.99
Decision Table	97.72	97.74	98.35
Decision Tree	97.41	97.76	98.95
Instance-based Learning	**99.99**	99.98	99.99
Multilayer Perceptron	99.84	99.88	99.93
Naive Bayes	84.15	85.76	92.78
SVM (linear kernel)	99.85	99.79	99.95
SVM (quadratic kernel)	99.75	99.80	99.95
SVM (cubic kernel)	99.72	99.80	99.91

**Table 2.** Comparison of the recognition accuracy (%) of classifying walking directions based on the raw data set (PIR_1_) with respect to the number of modules.

**Classifier**	**Recognition Accuracy** (%)						
	Module_1_ 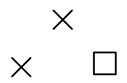	Module_2_ 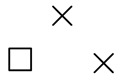	Module_3_ 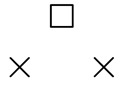	Module_1,2_ 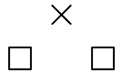	Module_1,3_ 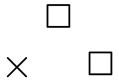	Module_2,3_ 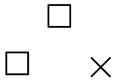	Module_1,2,3_ 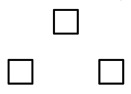
	PIR_1_ 	PIR_1_ 	PIR_1_ 	PIR_1_ 	PIR_1_ 	PIR_1_ 	PIR_1_ 
Bayes Net	99.51	98.58	95.43	**100**	99.41	99.75	99.99
Decision Table	93.39	93.24	95.51	95.50	95.61	97.22	97.72
Decision Tree	95.71	96.03	97.80	97.89	97.18	98.19	97.41
Instance-based Learning	**99.36**	**99.42**	**99.64**	**100**	99.63	99.61	99.99
Multilayer Perceptron	99.12	98.42	99.27	99.84	99.13	99.11	99.84
Naive Bayes	86.03	85.52	63.31	73.50	83.25	65.22	84.15
SVM (linear kernel)	93.23	92.00	74.12	99.76	98.49	98.61	99.85
SVM (quadratic kernel)	97.86	96.86	90.63	99.65	98.97	98.79	99.75
SVM (cubic kernel)	98.35	97.47	98.21	99.60	98.84	98.66	99.72

**Table 3.** Comparison of the recognition accuracy (%) of classifying walking distance intervals based on the raw data set with respect to the number of PIR sensors of all modules.

**Classifier**	**Recognition Accuracy** (%)		
	Module_1,2,3_ 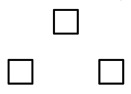		
	PIR_1_ 	PIR_1,3_ 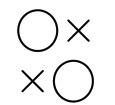	PIR_1.2,3,4_ 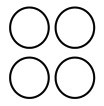
Bayes Net	**99.82**	99.86	99. 93
Decision Table	95.50	97.50	97.50
Decision Tree	97.68	98.72	98.78
Instance-based Learning	**99.52**	99.66	99.81
Multilayer Perceptron	97.81	99.48	99.61
Naive Bayes	95.00	96.88	97.41
SVM (linear kernel)	88.48	94.29	98.57
SVM (quadratic kernel)	99.13	99.56	99.62
SVM (cubic kernel)	99.47	99.70	99.76

**Table 4.** Comparison of the recognition accuracy (%) of classifying the walking distance intervals based on the raw data set (PIR_1_) with respect to the number of modules.

**Classifier**	**Recognition Accuracy** (%)						
	Module_1_ 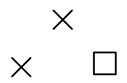	Module_2_ 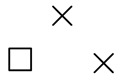	Module_3_ 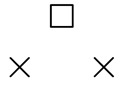	Module_1,2_ 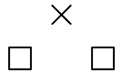	Module_1,3_ 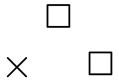	Module_2,3_ 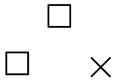	Module_1,2,3_ 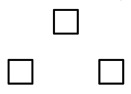
	PIR_1_ 	PIR_1_ 	PIR_1_ 	PIR_1_ 	PIR_1_ 	PIR_1_ 	PIR_1_ 
Bayes Net	75.56	69.44	95.39	73.46	99.48	98.22	98.82
Decision Table	72.95	70.45	92.27	78.73	93.82	94.92	95.50
Decision Tree	85.94	84.07	95.06	87.13	97.92	98.04	97.68
Instance-based Learning	81.92	79.63	**97.68**	**95.51**	97.89	96.04	**99.52**
Multilayer Perceptron	72.88	69.65	74.36	81.02	97.35	97.54	97.81
Naive Bayes	53.40	53.04	93.91	63.20	93.72	86.13	95.00
SVM (linear kernel)	46.27	44.75	45.69	59.08	71.47	86.78	88.48
SVM (quadratic kernel)	75.45	73.14	70.15	95.78	97.97	97.15	99.13
SVM (cubic kernel)	83.34	80.11	75.40	95.93	98.24	97.01	99.47

**Table 5.** Comparison of the recognition accuracy (%) of classifying walking speed levels based on the raw data set with respect to the number of PIR sensors of all modules.

**Classifier**	**Recognition Accuracy** (%)		
	Module_1,2,3_ 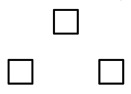		
	PIR_1_ 	PIR_l,3_ 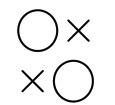	PIR_1,2,3,4_ 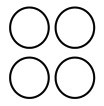
Bayes Net	77.93	78.34	79.11
Decision Table	76.59	76.83	77.08
Decision Tree	89.19	89.04	89.34
Instance-based Learning	**95.43**	95.72	95.87
Multilayer Perceptron	92.44	92.50	93.29
Naive Bayes	68.97	68.74	68.96
SVM (linear kernel)	81.25	82.38	84.76
SVM (quadratic kernel)	94.71	95.09	95.61
SVM (cubic kernel)	95.15	95.68	96.49

**Table 6.** Comparison of the recognition accuracy (%) of classifying walking speed levels based on the raw data set (PIR_1_) with respect to the number of modules.

**Classifier**	**Recognition Accuracy** (%)						
	Module_1_ 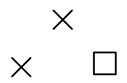	Module_2_ 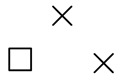	Module_3_ 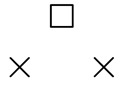	Module_1,2_ 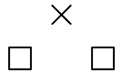	Module_1,3_ 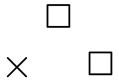	Module_2,3_ 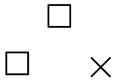	Module_1,2,3_ 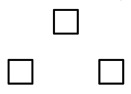
	PIR_1_ 	PIR_1_ 	PIR_1_ 	PIR_1_ 	PIR_1_ 	PIR_1_ 	PIR_1_ 
Bayes Net	67.31	70.24	78.94	75.83	77.31	77.77	77.93
Decision Table	72.49	75.29	76.08	75.35	74.98	75.50	76.59
Decision Tree	82.84	84.13	84.81	87.81	87.65	86.84	89.19
Instance-based Learning	79.34	80.36	**90.13**	**92.55**	90.19	89.15	**95.43**
Multilayer Perceptron	70.18	72.06	77.41	90.84	87.02	83.77	92.44
Naive Bayes	62.24	61.73	50.25	66.79	66.76	63.64	68.97
SVM (linear kernel)	50.63	58.95	56.99	71.87	74.67	71.87	81.25
SVM (quadratic kernel)	72.18	74.15	76.11	95.29	92.21	89.63	94.71
SVM (cubic kernel)	77.51	79.19	80.90	**94.63**	90.28	90.56	95.15

**Table 7.** Comparison of the recognition accuracy (%) of idengifying walking subjects based on the raw data set with respect to the number of PIR sensors of all modules.

**Classifier**	**Recognition Accuracy** (%)		
	Module_1,2,3_ 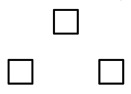		
	PIR_1_ 	PIR_l,3_ 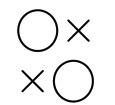	PIR_1,2,3,4_ 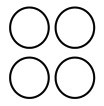
Bayes Net	49.90	56.88	63.74
Decision Table	48.18	52.87	51.41
Decision Tree	72.38	76.15	76.27
Instance-based Learning	**92.65**	**94.39**	**95.20**
Multilayer Perceptron	57.38	65.86	69.86
Naive Bayes	25.12	38.24	43.52
SVM (linear kernel)	68.12	77.27	82.77
SVM (quadratic kernel)	86.11	89.11	92.52
SVM (cubic kernel)	88.43	91.13	94.14

**Table 8.** Comparison of the recognition accuracy (%) of idengifying walking subjects based on the raw data set (PIR_1_) with respect to the number of modules.

**Classifier**	**Recognition Accuracy** (%)						
	Module_1_ 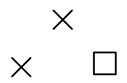	Module_2_ 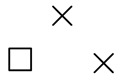	Module_3_ 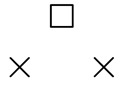	Module_1,2_ 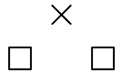	Module_1,3_ 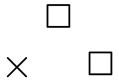	Module_2,3_ 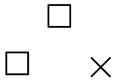	Module_1,2,3_ 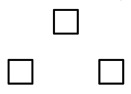
	PIR_1_ 	PIR_1_ 	PIR_1_ 	PIR_1_ 	PIR_1_ 	PIR_1_ 	PIR_1_ 
Bayes Net	35.86	41.11	26.84	50.60	41.95	43.77	49.90
Decision Table	35.41	37.40	28.40	48.91	42.98	44.88	48.18
Decision Tree	55.33	57.84	52.91	72.24	67.92	67.86	72.38
Instance-based Learning	59.55	55.09	63.07	**87.30**	77.43	74.59	**92.65**
Multilayer Perceptron	29.57	31.68	23.82	50.46	45.69	42.22	57.38
Naive Bayes	22.64	29.27	16.16	26.54	22.31	21.47	25.12
SVM (linear kernel)	24.19	26.52	14.87	51.65	41.25	40.53	68.12
SVM (quadratic kernel)	43.31	40.95	25.93	84.91	74.60	72.02	86.11
SVM (cubic kernel)	56.36	52.58	39.56	86.15	75.22	70.95	88.43

**Table 9.** Comparison of recognition accuracy (%) of classifying walking directions based on the reduced feature set with respect to the modules and features (amplitude and time to peaks).

**Classifier**	**Recognition Accuracy** (%)							
	Module_1_ 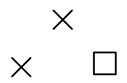		Module_2_ 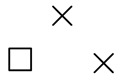		Module_3_ 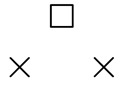		Module_1,2_ 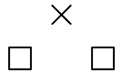	
	PIR_1_ 		PIR_1_ 		PIR_1_ 		PIR_1_ 	
	Amplitude	Amplitude and time	Amplitude	Amplitude and time	Amplitude	Amplitude and time	Amplitude	Amplitude and time
Bayes Net	97.50	97.77	96.15	97.27	77.77	92.68	99.19	99.23
Instance-based Learning	**96.94**	97.73	**97.25**	97.81	81.50	**94.02**	99.22	99.39
Multilayer Perceptron	97.57	98.31	97.69	98.10	76.56	93.12	99.45	99.45
SVM (linear kernel)	97.63	97.60	97.67	97.49	67.79	88.95	99.28	99.15

**Table 10.** Comparison of the recognition accuracy (%) of classifying walking distance intervals based on the reduced feature set with respect to the modules and features (amplitude and time to peaks).

**Classifier**	**Recognition Accuracy** (%)					
	Module_3_ 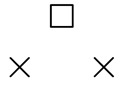		Module_1,2_ 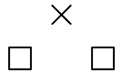		Module_1,2,3_ 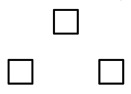	
	PIR_1_ 		PIR_1_ 		PIR_1_ 	
	Amplitude	Amplitude and time	Amplitude	Amplitude and time	Amplitude	Amplitude and time
Bayes Net	92.91	90.90	69.17	75.13	93.11	95.77
Instance-based Learning	**93.13**	90.40	74.96	**90.40**	89.97	**98.05**
Multilayer Perceptron	86.13	84.13	68.91	81.04	89.85	94.78
SVM (quadratic kernel)	69.21	74.84	68.10	84.90	89.30	96.84
SVM (cubic kernel)	69.59	75.39	69.65	87.63	90.32	97.60

**Table 11.** Comparison of the recognition accuracy (%) of classifying walking speed levels based on the reduced feature set with respect to the modules and features (amplitude and time to peaks and passage duration).

**Classifier**	**Recognition Accuracy** (%)					
	Module_3_ 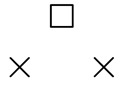		Module_1,2_ 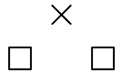		Module_1,2,3_ 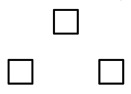	
	PIR_1_ 	PIR_1,2,3,4_ 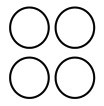	PIR_1_ 	PIR_1,2,3,4_ 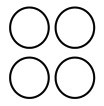	PIR_1_ 	PIR_1,2,3,4_ 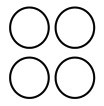
	Amplitude, time, and passage duration	Amplitude, time, and passage duration	Amplitude, time, and passage duration	Amplitude, time, and passage duration	Amplitude, time, and passage duration	Amplitude, time, and passage duration
Instance-based Learning	84.49	**90.54**	88.32	89.75	89.02	89.34
SVM (quadratic kernel)	74.50	86.13	85.11	**94.11**	86.29	**94.80**
SVM (cubic kernel)	78.34	**90.58**	87.07	**94.29**	89.62	**94.94**

**Table 12.** Comparison of the recognition accuracy (%) of idengifying walking subjects based on the reduced feature set with respect to the modules and PIR sensors.

**Classifier**	**Recognition Accuracy** (%)					
	Module_1,2_ 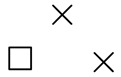			Module_1,2,3_ 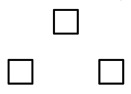		
	PIR_1_ 	PIR_1,3_ 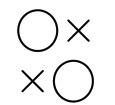	PIR_1,2,3,4_ 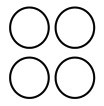	PIR_1_ 	PIR_1,3_ 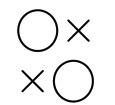	PIR_1,2,3,4_ 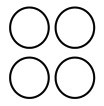
	Amplitude, time, and passage duration	Amplitude, time, and passage duration	Amplitude, time, and passage duration	Amplitude, time, and passage duration	Amplitude, time, and passage duration	Amplitude, time, and passage duration
Instance-based Learning	75.06	79.36	83.11	76.95	79.22	83.68
SVM (quadratic kernel)	59.09	79.86	**91.86**	72.10	88.88	**96.33**
SVM (cubic kernel)	66.08	83.24	**92.31**	77.41	89.47	**96.56**
